# Expression of Immunity- and Stress-Related Genes during an Intermolt Period in the Colorado Potato Beetle

**DOI:** 10.3390/insects13121168

**Published:** 2022-12-16

**Authors:** Vadim Yu. Kryukov, Ulyana N. Rotskaya, Olga N. Yaroslavtseva, Yury A. Noskov, Viktor V. Glupov

**Affiliations:** Institute of Systematics and Ecology of Animals, Siberian Branch of Russian Academy of Sciences, Frunze Str. 11, 630091 Novosibirsk, Russia

**Keywords:** ontogeny, immunity, Leptinotarsa, pathogen, mycosis

## Abstract

**Simple Summary:**

During ontogeny, many insects change habitats, which are characterized by specific communities of opportunistic and pathogenic microorganisms. Therefore, different developmental stages may be subject to different directions of natural selection that cause alterations in immunity. We analyzed changes in humoral immunity during the intermolt period of last-instar larvae of the Colorado potato beetle under laboratory conditions. We observed an increase in basal expression of immunity- and stress-related genes at the stages of finishing feeding and migration into soil. Changes in gene expression in response to fungal infection were also examined. The results suggest that the larvae develop strong defenses against soil pathogens (especially against fungi) before migration into soil. The study is also promising in terms of a gene knockdown for enhancing the susceptibility of the Colorado potato beetle to native or introduced fungal pathogens in potato agrosystems.

**Abstract:**

Different developmental stages of insects may be dissimilar in immunity functioning. Additionally, the stages often inhabit diverse environments with specific microbial communities. In the Colorado potato beetle, a strong increase in resistance to entomopathogenic fungi is observed during the intermolt period of last-instar larvae, but mechanisms of this change are insufficiently understood. We studied changes in the expression of immunity- and stress-related genes in the fat body and integument during this intermolt period by quantitative PCR. By the end of the instar, there was upregulation of transcription factors of Toll, IMD, and Jak–Stat pathways as well as genes encoding metalloprotease inhibitors, odorant-binding proteins, and heat shock proteins. Nonetheless, the expression of gene *LdRBLk* encoding β-lectin did not change during this period. Most of the aforementioned genes were upregulated in response to *Metarhizium robertsii* topical infection. The expression alterations were more pronounced in recently molted larvae than in finishing feeding larvae and in the integument compared to the fat body. We believe that upregulation of immune-system- and stress-related genes at the end of the intermolt period is an adaptation caused by migration of larvae into soil, where the probability of encountering entomopathogenic fungi is high.

## 1. Introduction

Insect immune reactions and susceptibility to pathogenic microorganisms change during ontogeny. It is well known that larvae of different instars, pupae, and adults demonstrate different levels of immunity and resistance to pathogens [1,2]. An important role in the formation of these reactions can be determined by metamorphosis and the habitat in which it occurs. Each habitat (e.g., a plant, leaf litter, or soil) contains a specific community of microorganisms, including pathogens. Habitats can be switched by insects within one larval instar. For holometabolous herbivores, this is often the last larval instar when the insects finish feeding on a plant and migrate into soil to pupate. Soil is the richest reservoir of microorganisms, including entomopathogenic fungi, capable of penetrating the host through the integuments. The density of entomopathogenic ascomycetes such as *Beauveria* and *Metarhizium* in soil reaches substantial concentrations: 10^4^–10^5^ colony-forming units per gram [3,4]. Infection of insects by these fungi in natural and anthropogenic ecosystems most often occurs in the leaf litter and soil [3]. From this point of view, changes in the host’s immunity at stages of completion of feeding and burrowing into the soil are of interest.

The Colorado potato beetle (CPB) *Leptinotarsa decemlineata* Say (Coleoptera, Chrysomelidae) is one of the most dangerous potato pests in the world. It is known that entomopathogenic fungi can have a significant impact on the natural regulation of its populations and are used for the inundative biological control of this pest (reviewed by [5,6]). Natural epizootics caused by fungi are extremely rare among feeding CPB larvae; however, high mortality from fungal infections can be observed at the stages inhabiting soil: the prepupa, pupa, and hibernating adults (reviewed by [7]). At the same time, the resistance to fungi at CPB soil stages is higher than in the feeding larvae. In particular, we have studied changes in susceptibility to entomopathogenic fungi during the intermolt period of the last larval instar [8] and observed a gradual increase in resistance to the fungi at the end of this period. During development within the last instar, along with thickening of the cuticle, there was a decrease in the level of adhesion of conidia to the cuticle, which correlated with a decrease in the amount of epicuticular hydrocarbons. In addition, during this period, significant elevation of the encapsulation activity and of the total hemocyte count was observed [8]. Thus, the change in habitats from a plant to soil is accompanied by an increase in cuticular and cellular defense systems associated with resistance to fungal infections. Nevertheless, alterations of molecular mechanisms such as regulation of immune signaling pathways during this period have remained unexplored.

Among insect immune signaling pathways, the most important are Toll, IMD, and Jak–Stat. The Toll cascade is related to defense against Gram-positive bacteria and fungi, whereas the IMD pathway is mainly related to defense against Gram-negative bacteria and Jak–Stat against viruses [9]. Nonetheless, IMD and Jak–Stat pathways are also upregulated in insects in response to fungal infections, and silencing or chemical inhibition of IMD and Jak–Stat cascades leads to increased susceptibility to fungi [10,11]. On the one hand, this phenomenon may indicate synergy between different pathways against fungal invaders. On the other hand, different bacteria may often be involved in the process of mycosis, thereby aggravating the pathogenesis and accelerating mortality (e.g., [12]).

Insect metalloproteinase inhibitors (IMPIs) are some of the key antifungal compounds inactivating the fungal proteinases [13]. Ricin-like β lectins (RBLs) are related to C-type lectins and may function in insect defenses against fungal infections [14] and against neonicotinoid insecticides [15]. Odorant-binding proteins (OBPs) function in a wide range of physiological processes and may change expression in response to fungal infections [16]. Heat shock proteins (HSPs) are stress-induced proteins. HSP70 and HSP90 are cochaperones and work as adaptors and folding proteins [17]. Expression of HSPs is dependent on insect development [18], and induction of HSPs in response to different infections, including a fungal invasion, was also reported (e.g., [19]). It should be noted that the research into activities of CPB immunity during development various pathologies is “far from exhaustive” [20]. At the same time, understanding the mechanisms that work against fungal pathogens in the CPB may allow for improved biological control of this pest.

In the present work, we studied changes in the expression of CPB genes related to immune signaling pathways (Toll, IMD, and Jak–Stat) as well as to the production of IMPIs, OBPs, HSPs, and C-type lectins in the fat body and integument during the intermolt period of the last larval instar. Changes in the expression of these genes in response to fungal infection (*Metarhizium robertsii)* at different stages of the period were also analyzed.

## 2. Materials and Methods

### 2.1. Insects and Fungi

Third-instar larvae of *L. decemlineata* were collected in potato kitchen gardens (*Solanum tuberosum*) near Karasuk town, Western Siberia (53°43′05.4″ N 77°38′14.4″ E). The larvae were maintained in a laboratory in 350 mL ventilated plastic containers (10 larvae per container) at 25 °C, 20% relative humidity, under a photoperiod of 14:10 light:dark. Foliage of *S. tuberosum* was used as feed.

Strain P-72 of *M. robertsii* (GenBank accession no. KP172147.2) was used, taken from the collection of microorganisms at the Institute of Systematics and Ecology of Animals, the Siberian Branch of the Russian Academy of Sciences. To obtain conidia, twice-autoclaved millet was inoculated with a 4-day-old submerged culture of P-72 and incubated for 10 days at 25 °C in the dark. Then, the culture was dried for 12 days at room temperature, and conidia were collected using a soil sieve and stored at 4 °C for 20 days until infection. For infecting the CPB, the conidia were suspended in an aqueous solution of Tween 20 (0.05%). Concentrations of the conidia were estimated by means of a Neubauer hemocytometer (Paul Marienfeld GmbH & Co. KG, Lauda-Königshofen, Germany).

### 2.2. The Infection Procedure and General Experimental Setup

Larvae of instar IV at 2 h post-molt (recently molted larvae) and of instar IV at 84 h post-molt (finishing feeding larvae) were collected for experiments. For infection, we used the immersion of larvae in a suspension of conidia for uniform distribution of the inoculum. The larvae were infected by immersion for 15 s in the aqueous Tween 20 suspension of *M. robertsii* (5 × 10^6^ conidia/mL, 1.5 mL per 10 larvae). In the control group, insects were treated with the conidia-free aqueous Tween 20 solution. Then, the larvae were maintained and fed as described in the previous section. Mortality assays were performed on eight replicates (1 replicate = 10 larvae) for 10 days.

For analysis of gene expression, we used control and infected insects of both groups: recently molted and finishing feeding larvae. Samples of the integument and fat body were taken 24 and 72 h after inoculation in each group. The analyses were performed separately for age-related changes (only uninfected larvae at 26, 108, and 156 h post-molt) and for a response to infection (control vs. infected insects of both groups: recently molted and finishing feeding larvae).

### 2.3. Sample Preparation and Quantitative PCR (qPCR)

Samples of the integument and fat body tissues were collected as described previously [14] with minor modifications. Integuments and fat bodies from three insects were pooled into one sample. The samples were collected into cold phosphate buffer on ice and frozen in liquid nitrogen. Long-term storage was implemented at −80 °C. Before RNA extraction, samples were lyophilized at −65 °C and 600 mtorr for 14 h and homogenized with micropestles in liquid nitrogen. Total RNA extraction was performed as described earlier [14]. DNase treatment was carried out according to the manual for the RQ1 RNase-free DNase Kit (Promega, Madison WI, USA). The reverse transcription of RNA to cDNA was performed with RevertedAid^TM^ M-MuLV Reverse Transcriptase (Fermentas, Vilnius, Lithuania) and 1.5 pmol of 9N primers.

qPCR was carried out by means of the HS-qPCR SYBR Blue (2×) mix (BioLabMix, Novosibirsk, Russia) on a thermal cycler called the CFX96 Touch Real-Time PCR detection system (Bio-Rad, Hercules, CA, USA). The qPCR was conducted in triplicate under the following conditions: 95 °C for 3 min, followed by 40 cycles of 94 °C for 15 s and annealing and elongation at 64 °C for 30 s, followed by melting curve analysis (70–90 °C). The melting curves for each sample were analyzed after each run to check the specificity of amplification. The following genes of *L. decemlineata* were used as a reference: 60S ribosomal protein L18 (*rp18*) and ADP-ribosylation factors 4 and 1 (*arf2* and *arf19*) [21]. The analyzed genes of interest were *dorsaldif*, *nfkb*, *stat*, *impi*, *LdRBLk*, *Ldobp*, *hsp70*, and *hsp90*. Genes’ full names and primer sequences are provided in Table 1. Primers were designed with the help of the NCBI Primers-BLAST resource [22], and primers’ properties were estimated in IDT OligoAnalyser 3.1 [23]; the primers were synthesized by the Biosset company (Novosibirsk, Russia).

Gene expression was calculated by the 2^ΔΔCq^ method in Bio-Rad CFX Manager (Bio-Rad). In the analysis of age-related changes, data on gene expression were normalized to those of larvae at 24 h post-molt. In the analysis of gene expression in response to infection, data were normalized to gene expression in uninfected larvae for each age group and each time point post-inoculation.

### 2.4. Statistics

Software packages PAST 3.0 [24] and SigmaStat 3.1 (Systat Software Inc., Tulsa, OK, USA) were used for the analyses. Because the data distribution was non-normal (according to the Shapiro–Wilk *W* test, *p* < 0.05), Kruskal–Wallis ANOVA followed by Dunn’s post hoc test was applied to determine the significance in differences (*p* < 0.05). The log rank test was performed to evaluate differences in survival dynamics. Data in plots are presented as the arithmetic mean and standard error.

## 3. Results

### 3.1. Susceptibility to M. robertsii

**Figure 1:**
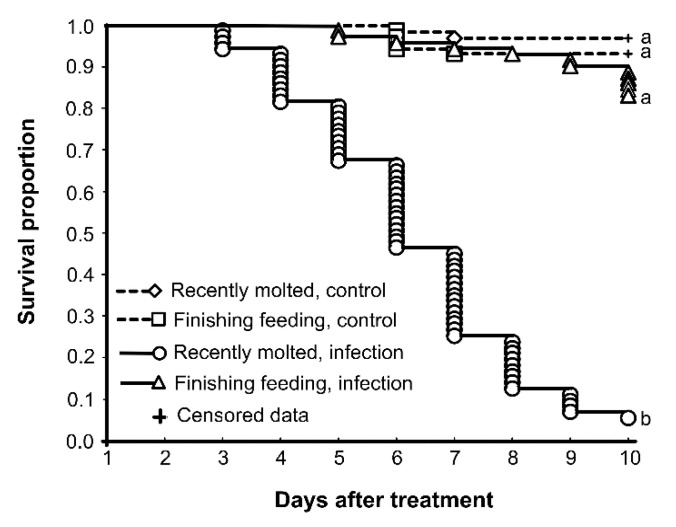
Survival of CPB larvae at different stages after topical infection with *M. robertsii* (5 × 10^6^ conidia/mL). Recently molted larvae were infected at 2 h post-molt in instar IV. Finishing feeding larvae were infected at 86 h post-molt in instar IV. Different letters show significant differences in survival dynamics (log rank test, χ^2^ > 110.3, df = 1, *p* < 0.0001).

We repeated the survival assay in the present study, and the results were consistent with previous work [8]. The infection of recently molted larvae led to a significant decrease in survival, down to 5% over 10 days (Figure 1). Infection of finishing feeding larvae led to 85% survival over 10 days; this result did not significantly differ from controls, which showed 93–97% survival.

### 3.2. Changes in Gene Expression during Development

**Figure 2:**
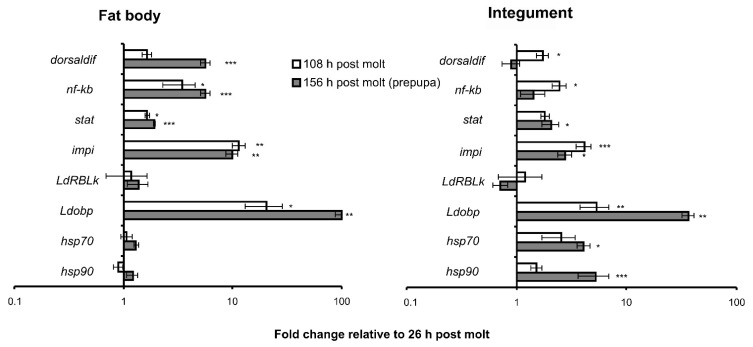
Changes in expression of immunity- and stress-related genes during the intermolt period in CPB larvae (instar IV, no infection). Data are presented as fold changes relative to uninfected larvae at 26 h post-molt. Genes *rp18*, *arf2*, and *arf19* were used as references. * *p* < 0.05, ** *p* < 0.01, and *** *p* < 0.001 relative to larvae at 26 h post-molt (Dunn’s test).

Transcription factors of Toll, IMD, and Jak–Stat immune signaling pathways (*dorsaldif, nfkb*, and *stat*) increased expression during the intermolt period (Figure 2). In particular, the upregulation of *dorsaldif, nfkb*, and *stat* in the fat body was time-dependent and reached 1.6–3.5-fold elevation in finishing feeding larvae and 2–6-fold elevation in prepupae compared to recently molted larvae. In the integument, significant upregulation of *dorsaldif* and *nfkb* was registered only in finishing feeding larvae (2–2.5-fold compared to recently molted larvae) but not in prepupae. *Stat* expression increased 1.8–2-fold in both finishing feeding larvae and prepupae compared to recently molted larvae, although significant differences were detected only in prepupae.

Expression of the *impi* gene also increased during larval development. The strongest upregulation was observed in the fat body, reaching 10–11-fold elevation in finishing feeding larvae and prepupae compared to recently molted larvae. In integument, this enhancement was 3–4-fold. *LdRBLk* gene expression did not significantly change as a function of larval stages in the fat body and integument. The *Ldobp* gene showed the strongest upregulation during the intermolt period: 21-fold and 100-fold in the fat body of finishing feeding larvae and prepupae, respectively, and 6-fold and 35-fold in the integument of finishing feeding larvae and prepupae compared to recently molted larvae, respectively. *Hsp70* and *hsp90* gene expression did not change during larval development in the fat body but significantly went up in the cuticle of the prepupa stage compared to recently molted larvae.

### 3.3. Alterations of Gene Expression in Response to M. robertsii Infection

**Figure 3:**
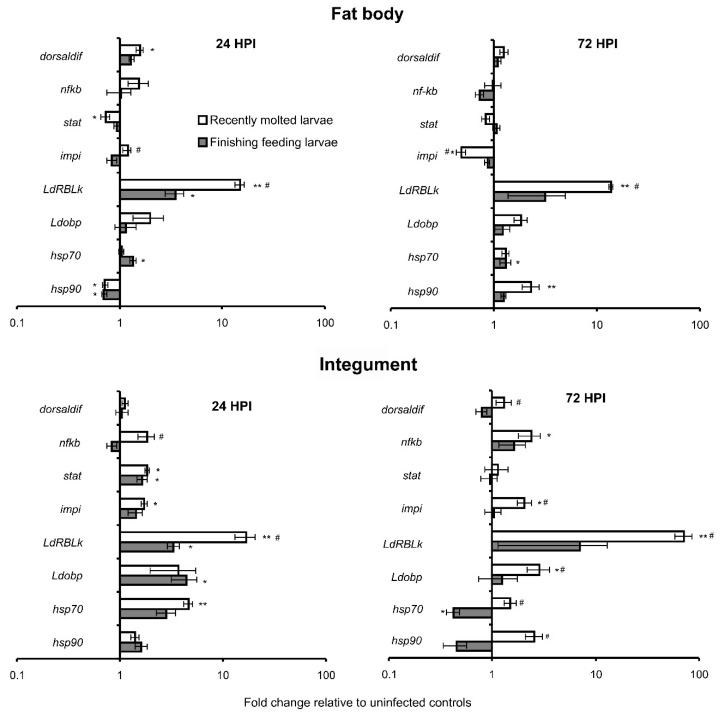
Alterations of expression of immunity- and stress-related genes in different stages of CPB larvae at 24 h post-inoculation (HPI) and 72 HPI with *M. robertsii* (5 × 10^6^ conidia/mL). Data are presented as fold changes relative to an uninfected control in each stage at each time point. Genes *rp18*, *arf2*, and *arf19* were used as references. * *p* < 0.05, ** *p* < 0.01 relative to uninfected controls (Dunn’s test). ^#^ Significant differences in expression between infected recently molted larvae and infected finishing feeding larvae (Dunn’s test, *p* < 0.05).

Differential expression of the studied genes was registered after topical infection of CPB larvae with *M. robertsii* (Figure 3). More pronounced responses were detected in recently molted larvae compared to finishing feeding larvae and in the integument compared to the fat body.

Expression of genes of transcription factors *dorsaldif, nfkb*, and *stat* showed minor changes in response to *M. robertsii* infection; nonetheless, some of these alterations were significant (Figure 3). In particular, *dorsaldif* gene expression significantly increased in the fat body at the initial stage of mycosis (24 h) in recently molted larvae (1.6-fold relative to uninfected control). *Nfkb* gene expression was found to be significantly upregulated only in the integument of recently molted larvae at 72 h after infection (2.4-fold relative to control). Notably, *nfkb* was upregulated in the integuments of recently molted larvae compared to finishing feeding larvae only at 24 h after treatment, and the same effect was registered for *dorsaldif* at 72 h after treatment. The *Stat* gene tended to be slightly downregulated in the fat body at 24 h after infection initiation, with a significant decrease in recently molted larvae. By contrast, the *stat* gene was upregulated in the integument in response to infection (1.7–1.8-fold) in both recently molted and finishing feeding larvae at 24 h post-inoculation. At the letter stage of mycosis (72 h), there were no significant changes in *stat* expression in response to infection in the fat body and integument of both larval stages.

The *impi* gene was downregulated in the fat body at 72 h in response to infection in recently molted larvae but upregulated in the integument of the same larvae at both time points 24 and 72 h post-inoculation (1.7–2.1-fold relative to controls). There were no significant alterations of *impi* gene expression in finishing feeding larvae under the influence of the fungal infection in both tissues and at both time points.

The *LdRBLk* gene was dramatically upregulated in response to the infection in both tissues and more strongly in recently molted larvae. In particular, 14–15-fold upregulation in the fat body of recently molted larvae was observed at 24 and 72 h post-inoculation, whereas weaker (3.2–3.5-fold) upregulation was registered in finishing feeding larvae at these time points. In the integument, the *LdRBLk* gene was found to be upregulated 16-fold and 72-fold at 24 and 72 h, respectively, in recently molted larvae but only 3–7-fold at the same time points in finishing feeding larvae.

The *Ldobp* gene tended to be upregulated in response to the fungal infection. A significant increase in its expression was detected in the integument at 24 h after the inoculation in finishing feeding larvae (4.4-fold relative to control) and at 72 h after the inoculation in recently molted larvae (2.9-fold relative to control).

*Hsp70* and *hsp90* manifested complicated, not straightforward changes in expression, depending on larval stages, time after infection, tissue, and the genes. *Hsp70* tended to be upregulated in the fat body in response to infection at both larval stages, but a significant increase was registered in finishing feeding larvae at 24 and 72 h (1.3–1.4-fold compared to control). In the integument, at 24 h after infection, the gene tended to be upregulated, and a significant increase was registered in recently molted larvae (4.6-fold relative to control). On the contrary, the gene was significantly downregulated at 72 h after infection in finishing feeding larvae (2.4-fold relative to control).

*Hsp90* was significantly downregulated in the fat body at 24 h post-inoculation at both larval stages (1.4–1.5-fold relative to controls) but upregulated at 72 h after infection in recently molted larvae (2.3-fold relative to control). In the integument, *hsp90* tended to be upregulated at 24 h post-inoculation at both larval stages, but the differences were not significant. At 72 h post-inoculation, *hsp90* (just as *hsp70*) tended to be downregulated in response to infection in finishing feeding larvae, but the differences were also not significant.

## 4. Discussion

This study shows that during the intermolt period of CPB last-instar larvae, there is an increase in the expression of genes related to Toll, IMD, and Jak–Stat immune signaling pathways as well as to IMPIs, OBPs, and HSPs in the fat body and/or integument. These findings coincided with an increase in resistance to the fungal pathogen *M. robertsii* and are also consistent with previously obtained data on the enhancement of cellular immunity and cuticular defenses during this period [8]. In response to a fungal infection, most of the studied genes changed expression (up- or downregulation). Larvae that were more susceptible to the fungus (recently molted) showed more pronounced gene expression than less susceptible larvae (finishing feeding), apparently due to thin and weakly sclerotized integuments. In addition, changes in expression in response to the infection were overall more pronounced in the integument than in the fat body.

The expression of transcription factors of Toll and IMD pathways (*dorsaldif* and *nfkb*) increased during the intermolt period, especially in the fat body. The role of the Toll pathway in the defense against fungal infections is well known (reviewed by [25]), although it is not known which antimicrobial peptides are the effectors of this pathway in the CPB. Notably, the Toll pathway may also control hemocyte proliferation and encapsulation [26,27]. This observation is consistent with the increase in hemocyte density and in encapsulation activity during this intermolt period, as shown in our previous study [8]. Upregulation of transcription factors of the IMD pathway in response to fungal infections has been demonstrated in dipteran insects [11,28,29]. Notably, IMD transcription factor *Rel2* is upregulated in antibiotic-treated insects in response to *Beauveria* and *Isaria* infections [11], probably indicating a specific response to a fungal infection but not to bacteria involved in the pathology. In our experiments, the upregulation of *dorsaldif* and *nfkb* in response to *M. robertsii* infection was rather weak (less than 2.4-fold relative to uninfected larvae) and was detected only in susceptible (recently molted) larvae. Nevertheless, we previously observed a significant 5–10-fold increase in the expression of these transcription factors in CPB larvae in response to topical infection with another fungus, *B. bassiana* (unpublished [30]). This fungus is the second most abundant species after the *Metarhizium* species in the soils of potato agrosystems [31]. Moreover, *Beauveria* is a leader in terms of the frequency of fungal infections in CPB populations [5,32]. We have also registered manifold stronger expression of the *attacin* gene (antimicrobial peptide of the IMD pathway) in the integument of CPB larvae in response to *B. bassiana* as compared to *M. robertsii* [14]. Probably, the level of *dorsaldif* and *nfkb* expression in CPB larvae depends on the fungus species and is more sensitive to the common pathogen *B. bassiana*.

A transcription factor of the Jak–Stat pathway (*stat*) showed an increase in expression in both tested tissues during the intermolt period in uninfected larvae as well as an increase in expression in the integument at the initial stages of *M. robertsii* infection (24 h post-inoculation). It has been shown that Jak–Stat cascade genes are expressed in response to fungal infections in lepidopterans and dipterans [10,11,28,33], and inhibition of the Jak–Stat pathway enhances the susceptibility of these insects to *B. bassiana* [10,33]. Accordingly, we can hypothesize that the overexpression of *dorsaldif*, *nfkb*, and *stat* during the intermolt period is associated with the formation of defenses against the fungal pathogens that are widespread in soil. It should be noted that during the last instar, the level of the 20-hydroxyecdysone titer increases, which enhances the activity of immuno-signaling cascades, and the production of antimicrobial peptides, as shown on *Helicoverpa armigera* [34]. Therefore, future studies may focus on the effect of 20-hydroxyecdysone on the induction of immunity in the Colorado potato beetle

The *impi* gene also increased expression at the end of the intermolt period in uninfected larvae. In response to the fungal infection, *impi* was upregulated mainly in the integument of susceptible (recently molted) larvae. It is known that IMPIs are produced as a reaction to fungal metalloproteinases, which contribute to the degradation of insect cuticle proteins [13]. It was shown on *Galleria mellonella* larvae that RNA interference-based silencing of *impi* leads to strong downregulation of its expression in the integument and less strong downregulation in the fat body, concurrently with an increase in susceptibility of the larvae to *Metarhizium brunneum* after topical infection [35]. In the present study, an increase in *impi* gene expression after the infection in recently molted larvae coincided with more severe damage and stronger melanization of their cuticle as compared to finishing feeding larvae [8]. The downregulation of *impi* in the fat body of the recently molted larvae is difficult to explain and may be related to the prioritization of immune responses between different tissues.

Expression of the gene encoding lectin LdRBLk did not change during the development of the insects under study but went up in response to the fungal infection in both tissues. The latter finding is in agreement with the results obtained earlier when the CPB was infected with *M. robertsii* and *B. bassiana* [14]. It is likely that this protein is inducible precisely in response to fungal infections but shows relatively stable basal expression in ontogeny. The strongest expression was observed here in susceptible (recently molted) larvae, especially in the integument. Thus, the expression of *LdRBLk* was symptomatic and corresponded to the level of lethality. It was previously reported that the structure of this protein allows it to have antifungal properties [14]. In particular, LdRBLk contains β-sheets that may interact with a fungal surface according to the carpet model [36], thereby resulting in destruction of the phospholipid bilayer of the membrane, followed by cell lysis.

We documented a significant increase in *Ldobp* gene expression during the intermolt period as well as its upregulation after the initiation of fungal infection. Wei et al. [16] registered upregulation of the *obp* gene mainly at 24–72 h post-inoculation by *B. bassiana* in the whole body of ants *Solenopsis invicta*. Those authors believe that OBPs are expressed mostly during the penetration of the cuticle by fungi. We also observed a significant increase in *Ldobp* expression only in the integument at 24 and 72 h post-inoculation. At this time point, *Metarhizium* usually does not yet circulate in the hemolymph of CPB larvae but is located in the integument and in encapsulated form under it [37]. It is noteworthy that in the larvae that are able to inactivate the fungal infection (finishing feeding), an increase in *obp* expression was observed at 24 h, whereas by 72 h, there was a decline to the control level. This result suggests that LdOBP can be considered a protein that exerts antifungal effects. This polypeptide contains the general odorant-binding protein domain (GOBP) [38]. Analysis of secondary structure of OBP from *L. decemlineata* by the self-optimized prediction method with alignment (SOPMA) revealed that it is a transmembrane polypeptide of 120 amino acid residues (aa) containing two transmembrane regions located within regions “aa 5–16” and “aa 54–56” (Figures S1 and S2). In these regions, the secondary structure of the protein folding is represented by a β-bridge, while the rest of the protein is folded into an α-helix interspersed by a random coil, consistent with previously described secondary structures of OBPs of insects [39]. This structure of the protein theoretically allows it to become integrated into the cell membrane of both insects and pathogens.

The expression of genes *hsp70* and *hsp90* increased at the end of larval development, but only in the integument. In response to the fungal infection, the genes also tended to be upregulated in the integument: in actively diseased (recently molted) larvae at 24 and 72 h post-inoculation and in resistant (finishing feeding) larvae only at 24 h post-inoculation. Previously, researchers also observed *hsps* upregulation as a reaction to fungal infections. For example, overexpression of *hsp90* has been recorded in *G. mellonella* in response to *B. bassiana* infection [40] and *Conidiobolus coronatus* infections [19]. In CPB larvae, there are trends toward an increase in *hsp90* expression in different tissues under the influence of *M. robertsii* and *B. bassiana* infections [14,41]. It is possible that induction of *hsps* expression in the integument during the intermolt period can enhance the defense against fungal infections because HSPs perform many general vitally important functions such as protein folding, unfolding, and localization, along with signaling and cell homeostasis [18]. Nonetheless, additional research is required to establish the role of HSPs in CPB ontogeny and immune responses.

## 5. Conclusions

This work uncovered changes in the expression of immune-system- and stress-related genes in CPB larvae during the intermolt period of last-instar larvae. Significant overexpression in the fat body and/or integument by the end of larval development was observed for transcription factors of immune signaling pathways (*dorsaldif*, *nfkb*, and *stat*) as well as for genes *impi*, *Ldobp*, *hsp70*, and *hsp90* but not for the gene encoding β-lectin, *LdRBLk*. In addition, most of these genes underwent an alteration of expression after topical application of *M. robertsii*, mainly upregulation. At the same time, the changes in the integuments were more pronounced than in the fat body. We propose that the higher expression of immunity- and stress-related genes by the end of the intermolt period is an adaptation associated with the migration of larvae into soil, where there is a high risk of contact with entomopathogenic fungi. This notion is in agreement with the enhancement of cellular immunity and integumental defense in CPB larvae during this period [8]. Future research projects may focus on silencing of the analyzed genes, thereby providing an opportunity to enhance CPB susceptibility to native or introduced pathogens in potato agrosystems.

## Figures and Tables

**Table 1:** The list and description of genes and primer sequences used in the qPCR.

Gene Name	GenBank Accession Number	Gene Symbol	Primer Sequence (5′→3′) *	Product Size (bp)	PCR Efficiency (± SD)
60S ribosomal protein L18	XM_023172940.1	*rp18*	For TAGAATCCTCAAAGCAGGTGGC Rev CTGGACCAAAGTGTT CACTGC	133	1.98 ± 0.02
ADP-ribosylation factor-like protein 4	KC190027.1	*arf2*	For GTGCTCGCGAACCATGTGA Rev AAACCTCCAATCCCTCGTGAAG	140	1.94 ± 0.01
ADP-ribosylation factor-like protein 1	XM_023169879.1	*arf19*	For CGGTGCTGGTAAAACGACAATATT Rev TGACCTCCCAAATCCCAAACTT	135	1.98 ± 0.02
DorsalDif-like protein	XM_023158121.1	*dorsaldif*	For TGTGCGAAAAGGTGGCTAAAG Rev ACTTGGGAGGGTTGGAAGTC	94	1.95 ± 0.05
NF-kappaB-like protein	XM_023174540.1	*nfkb*	For AAGCAGCGGTTTGATTCGTTC Rev AACTCGTCCAAGTTCTCCAGG	120	1.96 ± 0.06
Signal transducer and activator of transcription protein	XM_023165198.1	*stat*	For AGGAGCAGAACACAGGGTAC Rev TTTGCCTGGGAATTCTGTTGAC	140	1.94± 0.03
Inducible metalloproteinase inhibitor protein	XM_023167894.1	*impi*	For GCACCCTGTACAAGACCGT Rev CCGTGCGTAACCAGGTATACA	138	1.94 ± 0.02
Ricin-like β-lectin k	GEEF01084863.1	*LdRLBLk*	For GAACTGTTGATTGTCGTCACCATG Rev TGGAAAGTTTGGGAGATGGAACT	140	1.96 ± 0.04
Odorant binding protein	XM_023171367.1	*Ldobp*	For AGGTGAATCATGAAGTGCATTGC Rev GTGGTACCTCTCTGCTTCCTC	96	1.93 ± 0.02
Heat shock protein 70	KC544268.1	*hsp70*	For ACCCCGAAGAAGTCAGCTC Rev TCCAGGCCGTAAGCGATG	214	1.96 ± 0.04
Heat shock protein 90	KC556802.1	*hsp90*	For GGGTGTAGTCGACTCTGAAGAC Rev AGAGCTCCTCAAACAGTTCCAA	125	1.89 ± 0.01

* Primer sequences for *Rp18*, *Arf2*, and *Arf19* were taken from Shi et al. [21] and modified by Ulyana N. Rotskaya; the other primers were designed by Ulyana N. Rotskaya.

## Data Availability

The raw data of this article will be made available by the authors, without restrictions.

## Supplementary Materials

The following are available online at https://www.mdpi.com/article/10.3390/insects13121168/s1, Figure S1: The predicted secondary structure of *L. decemlineata* OBP (LdOBP) by Self-Optimized Prediction Method with Alignment (*SOPMA*) method [42], Figure S2: The predicted transmembrane regions of *L. decemlineata* OBP (LdOBP) by the Dense Alignment Surface (DAS) method [43].
