# Supplementary material for: Expression of Immunity- and Stress-Related Genes during an Intermolt Period in the Colorado Potato Beetle
Source: Insects. 2022 Dec 16;13(12):1168. doi: 10.3390/insects13121168 (PMC9785908; doi:10.3390/insects13121168)
Supplement: Supplementary file 1 [file insects-13-01168-s001.zip › insects-2054187-supplementary.pdf]

ELECTRONIC SUPPLEMENARY MATERIAL

Expression of immunity- and stress-related genes during an intermolt period in the Colorado potato beetle

Vadim Yu. Kryukov*, Ulyana N. Rotskaya, Olga N. Yaroslavtseva, Yuri A. Noskov, Viktor V. Glupov

Institute of Systematics and Ecology of Animals, Siberian Branch of Russian Academy of Sciences, Frunze Str. 11, Novosibirsk 630091, Russia

* Correspondance: krukoff@mail.ru


```
        10        20        30        40        50        60        70
         |         |         |         |         |         |         |
MKCIAVFVVVILALAHASESDREEAERYHLECQKDPASRIGEGAGPNSFAAHTLCVGVKMGTFKPNGDVD
hhhhhhheeeehhhhhhhhhhhhhhhhhhhhcccccccccchhhhttcccchheeeeeeeeeeccttcccc
VEGLRKFLIRTGVDPLKIDRIVHECSKRNGVTAVEAAMGMMGCLAKWTAT
hhhhhhhhhhhccchhhhhhhhhhhcccccccchhhhhhhhhhhhhhhhh
Sequence length :   120
SOPMA :
   Alpha helix      (Hh) :    71 is  59.17%
   3₁₀ helix        (Gg) :     0 is   0.00%
   Pi helix         (Ii) :     0 is   0.00%
   Beta bridge      (Bb) :     0 is   0.00%
   Extended strand  (Ee) :    14 is  11.67%
   Beta turn        (Tt) :     4 is   3.33%
   Bend region      (Ss) :     0 is   0.00%
   Random coil      (Cc) :    31 is  25.83%
   Ambiguous states (?)  :     0 is   0.00%
   Other states          :     0 is   0.00%
```

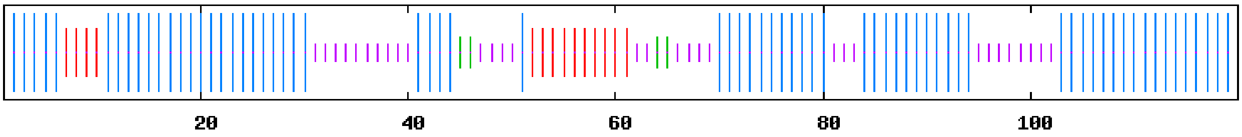

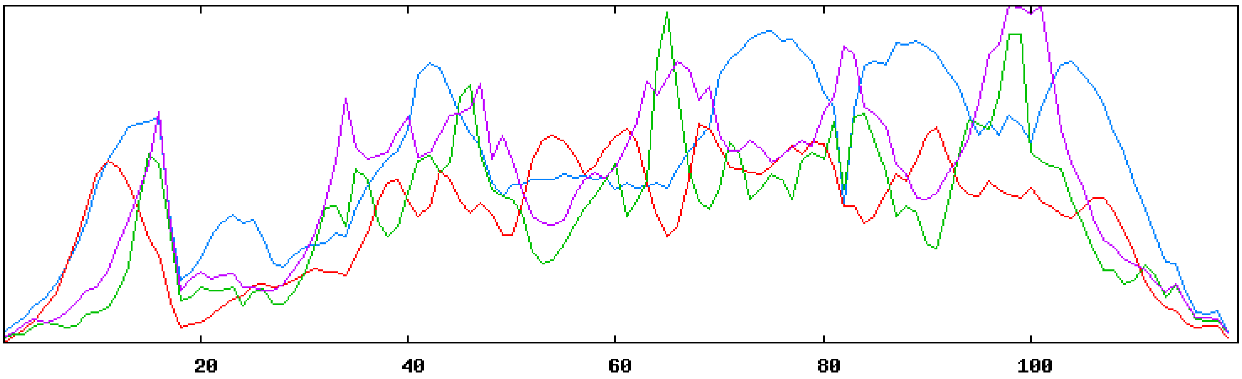

Figure S1. The predicted secondary structure of *L. decemlineata* OBP (LdOBP). The structure analysis of uncharacterized protein LOC111515132 [Leptinotarsa decemlineata] NCBI reference sequence XP_023027135.1 was made by Self-Optimized Prediction Method with Alignment (*SOPMA*) [42]. Parameters: window width: 17; similarity threshold: 8; number of states: 4.

```
Your query is:
MKCIAVFVVVILALAHASESDREEAERYHLECQKDPASRIGEGAGPNSFAAHTLCVGVKMGTFKPNGDVDVEGLRKF
LIRTGVDPLKIDRIVHECSKRNGVTAVEAAMGMMGCLAKWTAT
```

Potential transmembrane segments

Start	Stop	Length	~	Cutoff
5	15	11	~	2.2
5	16	12	~	1.7
54	56	3	~	1.7

The DAS curve for your query:

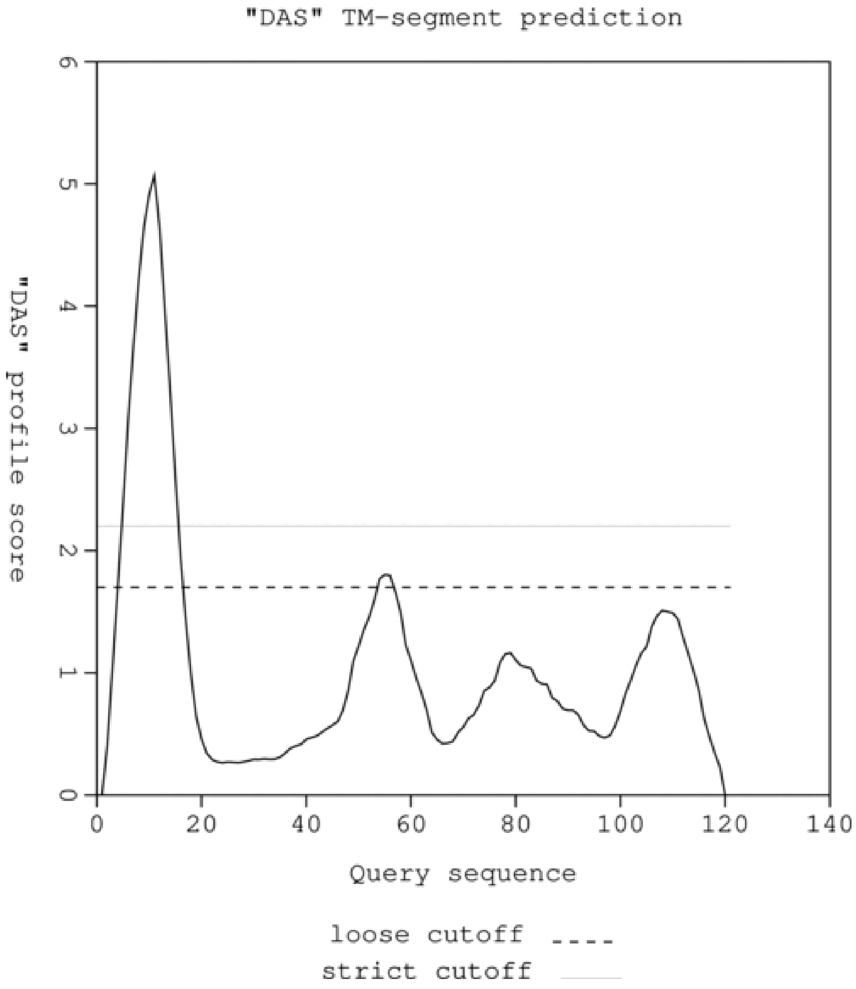

Figure S2. The predicted transmembrane regions of *L. decemlineata* OBP (LdOBP) by the Dense Alignment Surface method (DAS) [43]. These curves are obtained by pairwise comparison of the proteins in the test set in "each against the rest" fashion. There are two cutoffs indicated on the plots: a "strict" one at 2.2 DAS score, and a "loose" one at 1.7. The hit at 2.2 is informative in terms of the number of matching segments, while a hit at 1.7 gives the actual location of the transmembrane segment. The segments reported in the "FT" records of the SwissProt database are marked at 1.0 DAS score ("FT lines").

Supplementary references:

42. Geourjon, C.; Deléage, G.; SOPMA: Significant improvement in protein secondary structure prediction by consensus prediction from multiple alignments. *Cabios* **1995**, *11*, 681-684.

43. Cserzo, M.; Wallin, E.; Simon, I.; von Heijne, G.; Elofsson, A. Prediction of transmembrane alpha-helices in procariotic membrane proteins: the Dense Alignment Surface method. *Prot Eng* **1997**, *10*, 673-676.
